# Pf8: an open dataset of *Plasmodium falciparum *genome variation in 33,325 worldwide samples

**DOI:** 10.12688/wellcomeopenres.24031.1

**Published:** 2025-06-30

**Authors:** Malaria Genomic Epidemiology Network (MalariaGEN), Muzamil Mahdi Abdel Hamid, Mohamed Hassan Abdelraheem, Desmond Omane Acheampong, Ishag Adam, Pedro Aide, Olusola Ajibaye, Mozam Ali, Jacob Almagro-Garcia, Alfred Amambua-Ngwa, Lucas Amenga-Etego, Ifeyinwa Aniebo, Enoch Aninagyei, Felix Ansah, Tobias O Apinjoh, Cristina V Ariani, Sarah Auburn, Gordon A Awandare, Andrew Balmer, Philip Bejon, Simone Boene, George Bwire, Baltazar Candrinho, Arlindo Chidimatembue, Keobouphaphone Chindavongsa, Kiba Comiche, David Conway, Antoine Dara, Mahamadou Diakite, Abdoulaye Djimde, Arjen Dondorp, Seydou Doumbia, Eleanor Drury, Caterina A Fanello, Mike Ferdig, Katherine Figueroa, Dionicia Gamboa, Lemu Golassa, Sónia Gonçalves, Merepen dite Agnes Guindo, Mainga Hamaluba, Borimas Hanboonkunupakarn, Kevin Howe, Maazza Hussien, Mallika Imwong, Deus Ishengoma, Julia Jeans, Alinune Kabaghe, Appolinary Kamuhabwa, Jean-Marie Kindermans, Drissa S Konate, Dominic P Kwiatkowski, Chiyun Lee, Samuel K Lee, Sue J Lee, Benedikt Ley, Alejandro Llanos-Cuentas, Jutta Marfurt, Glória Matambisso, Rapeephan Rattanawongnara Maude, Richard James Maude, Alfredo Mayor, Mayfong Mayxay, Oumou Maïga-Ascofaré, Robert S McCann, Alistair Miles, Olivo Miotto, Abdelrahim Osman Mohamed, Collins Misita Morang’a, Kathryn Murie, Billy Ephraim Ngasala, Thuy-Nhien Nguyen, Oscar Nolasco, Francois Nosten, Rintis Noviyanti, Ísla O'Connor, Mary Oboh, Lynette Isabella Ochola-Oyier, Catherine Olufunke Falade, Adeola Olukosi, Ajibola Olumide, Fiyinfoluwa I Olusola, Marie A Onyamboko, Eniyou Cheryll Oriero, Wellington Aghoghovwia Oyibo, Danielle Pannebaker, Richard D Pearson, Kamija Phiri, Rob W van der Pluijm, Ric N Price, Huynh Hong Quang, Vinoth Rajkumar Devaraju, Milijaona Randrianarivelojosia, Lisa Ranford-Cartwright, Julian C Rayner, Eduard Rovira-Vallbona, Katherine Rowlands, Valentin Ruano-Rubio, Juan F Sanchez, Francisco Saúte, Shuwaram Shettima, Clemente da Silva, Victoria J Simpson, Simon Suddaby, Willem Takken, Aung Myint Thu, Mahamoudou Toure, Eyyub Unlu, Hugo O Valdivia, Michele van Vugt, Naomi Waithira, Thomas Wellems, Jason Wendler, Nina White, Rachel Wuendrich Ogidan

**Affiliations:** 1 Institute of Endemic Diseases, University of Khartoum, Khartoum, Sudan; 2 Nuclear Applications in Biological Sciences, Sudan Atomic Energy Commission, Khartoum, Sudan; 3 Department of Biomedical Sciences, School of Allied Health Sciences, University of Cape Coast, Cape Coast, Ghana; 4 University of Khartoum, Khartoum, Sudan; 5 Centro de Investigacao em Saude de Manhica, Manhica, Maputo Province, Mozambique; 6 Nigerian Institute of Medical Research, Lagos, Nigeria; 7 Wellcome Sanger Institute, Hinxton, England, UK; 8 Genomic Surveillance Unit, Wellcome Sanger Institute, Hinxton, England, UK; 9 Medical Research Council Unit The Gambia at the London School of Hygiene and Tropical Medicine, Banjul, The Gambia; 10 West African Centre for Cell Biology of Infectious Pathogens (WACCBIP), University of Ghana, Legon, Ghana; 11 Navrongo Health Research Centre, Navrongo, Ghana; 12 Health Strategy and Delivery Foundation, Abuja, Nigeria; 13 Department of Biomedical Sciences, School of Basic and Biomedical Sciences, University of Health and Allied Sciences, Ho, Ghana; 14 University of Buea, Buea, Cameroon; 15 Menzies School of Health Research, Charles Darwin University, Darwin, Northern Territory, Australia; 16 Nuffield Department of Medicine, University of Oxford, Oxford, England, UK; 17 KEMRI-Wellcome Trust Research Programme, Nairobi, Kenya; 18 University of Oxford, Oxford, England, UK; 19 Department of Pharmaceutical Microbiology, Muhimbili University of Health and Allied Sciences, Dar es Salaam, Tanzania; 20 National Malaria Control Programme, Maputo, Mozambique; 21 Center of Malariology, Parasitology and Entomology, Vientiane, Lao People's Democratic Republic; 22 London School of Hygiene & Tropical Medicine, London, England, UK; 23 University of Science, Techniques and Technologies of Bamako, Bamako, Mali; 24 Malaria Research and Training Centre, University of Science, Techniques and Technologies of Bamako, Bamako, Mali; 25 University Clinical Research Center, Bamako, Mali; 26 Mahidol Oxford Tropical Medicine Research Unit, Bangkok, Thailand; 27 University of Oxford Centre for Tropical Medicine and Global Health, Oxford, England, UK; 28 Department of Biological Sciences, University of Notre Dame, Notre Dame, Indiana, USA; 29 Laboratorio de Malaria: Parasitos y vectores, Laboratorios de Investigacion y Desarrollo, Facultad de Ciencias e Ingenieria, Universidad Peruana Cayetano Heredia, Lima, Peru; 30 Aklilu Lemma Institute of Pathobiology, Addis Ababa University, Addis Ababa, Ethiopia; 31 International Center for Excellence in Research, Faculty of Medicine and Odontostomatology, University of Sciences, Techniques and Technologies of Bamako, Bamako, Mali; 32 Mahidol University Faculty of Tropical Medicine, Bangkok, Thailand; 33 Mahidol University, Bangkok, Thailand; 34 National Institute for Medical Research, Dar es Salaam, Tanzania; 35 Kamuzu University of Health Sciences, Blantyre, Malawi; 36 Department of Clinical Pharmacy and Pharmacology, Muhimbili University of Health and Allied Sciences, Dar es Salaam, Tanzania; 37 Médecins Sans Frontières Operational Center, Brussels, Belgium; 38 The Broad Institute of Harvard and MIT, Cambridge, MA, USA; 39 Universidad Peruana Cayetano Heredia, Lima, Peru; 40 Department of Medicine, Mahidol University Faculty of Medicine Ramathibodi Hospital, Bangkok, Thailand; 41 Harvard University T H Chan School of Public Health, Boston, Massachusetts, USA; 42 ISGlobal, Barcelona, Spain; 43 Lao-Oxford-Mahosot Hospital-Wellcome Trust Research Unit, Microbiology Laboratory, Mahosot Hospital, Vientiane, Lao People's Democratic Republic; 44 Institute of Research and Education Development, University of Health Sciences, Ministry of Health, Vientiane, Lao People's Democratic Republic; 45 Bernhard Nocht Institute of Tropical Medicine, Hamburg, Germany; 46 Research in Tropical Medicine, Kwame Nkrumah University of Science and Technology, Kumasi, Ghana; 47 Center for Vaccine Development and Global Health, University of Maryland School of Medicine, Baltimore, Maryland, USA; 48 University of Khartoum Faculty of Medicine, Khartoum, Sudan; 49 EPPIcenter Research Program, Division of HIV, Infectious Diseases, and Global Medicine, University of California San Francisco Department of Medicine, San Francisco, California, USA; 50 Department of Parasitology and Medical Entomology, Muhimbili University of Health and Allied Sciences, Dar es Salaam, Tanzania; 51 Oxford University Clinical Research Unit, Ho Chi Minh City, Vietnam; 52 Shoklo Malaria Research Unit, Mahidol Oxford Tropical Medicine Research Unit Faculty of Tropical Medicine Mahidol University, Mae Sot, Thailand; 53 Eijkman Institute for Molecular Biology, Central Jakarta, Jakarta, Indonesia; 54 Faculty of Basic and Clinical Sciences, University of Ibadan College of Medicine, Ibadan, Nigeria; 55 First Technical University, Ibadan, Nigeria; 56 Department of Pharmacology & Therapeutics, University of Ibadan, Ibadan, Nigeria; 57 Kinshasa School of Public Health, University of Kinshasa, Kinshasa, Democratic Republic of the Congo; 58 The Kinshasa Medical Oxford Research Unit, Kinshasa, Democratic Republic of the Congo; 59 University of Lagos College of Medicine, Lagos, Nigeria; 60 US Naval Medical Research Unit SOUTH, Parasitology Department, Lima, Peru; 61 Infectious Disease Epidemiology and Analytics G5 Unit, Institut Pasteur Université Paris Cité, Paris, France; 62 Institute of Malariology, Parasitology, and Entomology (IMPE), Ministry of Health Vietnam, Quy Nhon, Vietnam; 63 Unité de Parasitologie, Institut Pasteur de Madagascar, Antananarivo, Madagascar; 64 Faculté des Sciences, Université de Toliara, Toliara, Madagascar; 65 School of Biodiversity, One Health and Veterinary Medicine, University of Glasgow College of Medical Veterinary and Life Sciences, Glasgow, Scotland, UK; 66 Cambridge Institute for Medical Research, University of Cambridge, Cambridge, UK; 67 Modibbo Adama University Teaching Hospital, Yola, Nigeria; 68 Department of Entomology, Wageningen University & Research, Wageningen, The Netherlands; 69 Academic Medical Center, University of Amsterdam, Amsterdam, The Netherlands; 70 Laboratory of Malaria & Vector Research (LMVR), National Institute of Allergy and Infectious Diseases Division of Intramural Research National Institutes of Health, Maryland, USA; 71 Seattle Children's Hospital, Seattle, Washington, USA

**Keywords:** malaria, Plasmodium falciparum, genomics, data resource, genomic epidemiology, genomic surveillance, open data sharing

## Abstract

We describe the Pf8 data resource, the latest MalariaGEN release of curated genome variation data on over 33,000 *Plasmodium falciparum* samples from 99 partner studies and 122 locations over more than 50 years. This release provides open access to raw sequencing data and genotypes at over 12 million genomic positions. For the first time, it includes copy-number variation (CNV) calls in the drug-resistance associated genes *gch1* and *crt*. As in Pf7, CNV calls are provided for *mdr1* and *plasmepsin2/3*, along with calls for deletion in *hrp2* and *hrp3,* genes associated with rapid diagnostic test failures. This data resource additionally features derived datasets, interactive web applications for exploring patterns of drug resistance and variation in over 5,000 genes, an updated Python package providing methods for accessing and analysing the data, and open access analysis notebooks that can be used as starting points for further analyses. In addition, informative example analyses show contrasting profiles of the decline of chloroquine resistance-associated mutations in Africa, and variation in copy number variation across 10 distinct sub-populations. To the best of our knowledge, Pf8 is the largest open data set of genome variation in any eukaryotic species, making it an invaluable foundational resource for understanding evolution, including that of pathogens.

## Background

Openly-available and high-quality genomic data are crucial contemporary components which support efforts to manage, control, and cure disease. The aggregation of genomic data across space and time enables surveillance and furthers our understanding of how disease-causing or disease-transmitting species evolve and spread. Such information can be used to discover and monitor epidemiologically-relevant genetic markers, to characterise and trace outbreaks, and evaluate the effectiveness and evolutionary impact of control measures ^1– 3^.

Genomic data has proven particularly relevant in tracking endemic diseases such as malaria, which remains a major public health burden ^4, 5^. In the latest annual World Malaria Report, the World Health Organisation (WHO) highlighted an increase in global malaria case numbers from the previous year and no progress in the reduction of deaths due to malaria ^4^. There were an estimated 263 million cases of malaria in 2023, of which 94% occurred in African countries. Malaria caused 597,000 deaths in 2023, most of which were caused by the species *P. falciparum.* Major challenges for malaria control include the rise of artemisinin partial resistance (ART-R) in Africa, the emergence of resistance to partner drugs used in artemisinin combination therapies (ACTs), and failure of rapid diagnostic tests (RDTs) due to *hrp2/3* deletions ^4^. These threats require robust, accessible, and interpretable genomic data in order to be addressed. Thus, it is vital to maintain whole genome resources for the community.

The Malaria Genomic Epidemiology Network (MalariaGEN) has been curating contributed whole genome data resources for the two major malaria parasite species *Plasmodium falciparum* and *P. vivax* ^6, 7^. The utility of MalariaGEN’s data releases is demonstrated via their widespread use, which has enabled research across the globe ( https://www.malariagen.net/parasite-observatory/#research). Diverse examples of recent works using MalariaGEN *P. falciparum* data resources include the identification of new gene mutations linked to chloroquine resistance ^8^; understanding variation in candidate drug target genes ^9^; determining the geographic origin of drug-resistant parasites entering the USA ^10^ and the likely historical spread of malaria to the Americas ^11^; identification of patterns of connectivity in Africa ^12^; developing new methods for analysing *var* gene expression ^13^; training machine learning models to understand the effects of non-coding variation ^14^; the discovery of a novel *P. falciparum* cryptotype ^15^; identifying variation in vaccine candidates ^16, 17^ and the binding sites of promising monoclonal antibodies ^18^; providing an increased understanding of *P. falciparum*’s interaction with both vector and human immune systems ^19, 20^; and informing theoretical models of transmission dynamics ^21^.

We now present Pf8, the latest global dataset of 33,325 *Plasmodium falciparum* genomes. Pf8 represents a significant advancement in the scale of openly available whole genome data for malaria parasites, expanding the size of the previous release, Pf7, by ~60%. Analyses included in the previous release, such as assessment of sample diversity, genetic structure, and prediction of drug resistance phenotypes are provided. New features in Pf8 include the addition of genomes collected in the 1960’s as well as since 2018, expanding the longitudinal scale available for analysis, and copy number variation (CNV) analysis for the genes *gch1* and *crt*. Additionally, two new breakpoints for *hrp3* are identified in Pf8. Finally, we include a suite of open access interactive applications and analysis tools to enhance the accessibility of the Pf8 data release.

## Resource data

Here we describe the Pf8 data resource. We intend that this serves as a high-level description of the data, rather than a comprehensive analysis of the release. We have presented select analyses as illustrative examples to highlight the breadth and diversity of the data. We encourage users of Pf8 to further co-develop analyses of this rich data resource particularly in collaboration with partner study leads to provide additional insights into malaria genomic epidemiology. In later sections we describe additional tools to aid in the exploration of Pf8 for such analyses.

### Sample collection

**Figure 1.**
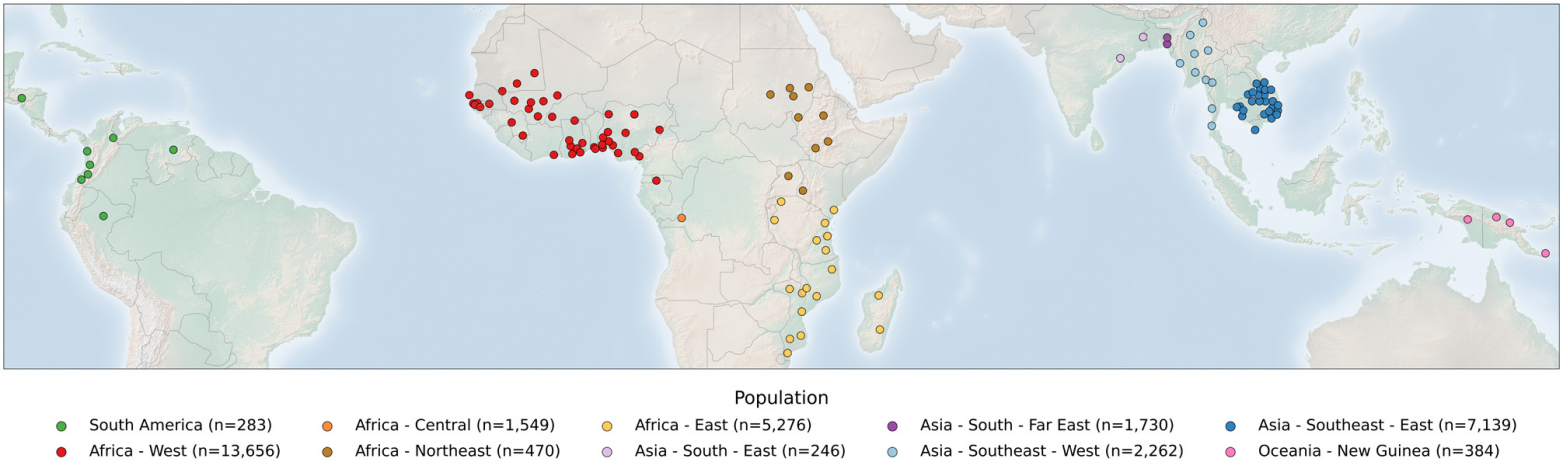
Global map of sample collection locations. The 122 first-level administrative divisions contained in Pf8 are represented, with points situated at the centre of each administrative division. Colours represent the ten subpopulations structuring the dataset.

**Figure 2.**
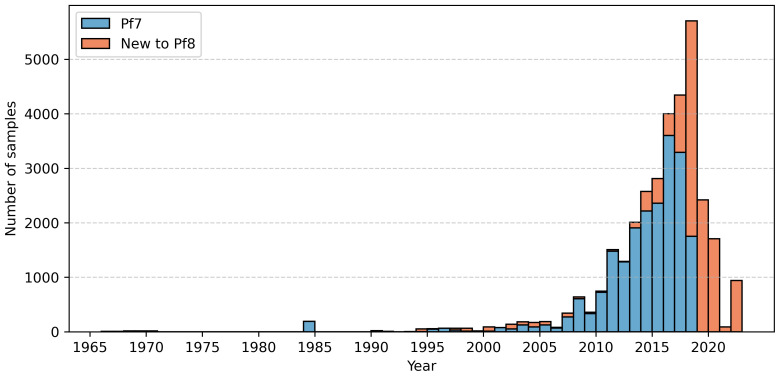
Sample collection over time. The blue bars represent samples collected for the previous *Plasmodium falciparum* data release, Pf7. The red bars represent samples newly collected for Pf8.

The Pf8 dataset contains 33,325 whole genome samples, collected from 122 first-level administrative divisions in 34 countries in South America, Africa, Asia, and Oceania ( Figure 1, Table 1). Pf8 contains both clinical field samples and samples originating from lab strains. Lab strain samples include crosses between, or mixtures of, different lab strains. Compared to the previous release, Pf7 ^7^, 12,461 samples have been added, including new field samples from Cambodia, the Democratic Republic of the Congo, Gambia, Ghana, Honduras, Kenya, Laos, Malawi, Mali, Mozambique, Nigeria, Peru, Sudan, Tanzania, Thailand, and Vietnam (Supplementary Figure 1). Samples in Pf8 were collected between 1966 – 2022, expanding the range of years represented by 22 years ( Figure 2) in comparison to Pf7. Sixty-one of these new samples were collected prior to 1984, while 2,733 (~8%) of the dataset are new samples from 2019–2022. Pf8 contains data from 99 partner studies, 17 more than in Pf7 (Supplementary Table 1). We applied quality control (QC) to individual samples, removing those with unverified or incomplete sample collection information (including lab strains), those with evidence of mixed infections with other *Plasmodium* species, duplicate samples from the same patient, samples with low coverage, and samples with a high number of singleton variants (evidence of being a significant genetic outlier). A total of 24,409 high-quality samples remained.

### Genomic variants

All Pf8 samples were processed through an analysis pipeline to produce genome-wide variant calls as described in Methods and Supplementary Methods. A key difference from the previous Pf7 pipeline is that parameters were changed to prefer single nucleotide polymorphisms (SNPs) over short indels where there are haplotypes that can be resolved as different combinations of variant types, and resolving using SNPs only makes downstream analyses more straightforward. For example, in Pf7, the CVIET haplotype at *crt*_72–76_ was represented by a single base pair insertion, a single base pair deletion and one SNP, whereas in Pf8 it is represented by four SNPs.

Across the entire 23MB *P. falciparum* genome, we discovered genomic variations in over half of all positions (12,493,205 variants) relative to the 3D7 v3 reference genome (Supplementary Table 2). Approximately one third of these positions (4,411,457) were SNPs. The majority of variable positions were non-SNPs, meaning 8,081,748 positions were short indels or SNP/indel combinations.

Variants were annotated and filtered according to QC thresholds described in the Methods. We excluded variants in the subtelomeric and internal hypervariable, mitochondrial, and apicoplast regions from passing variant QC. A total of 3,019,934 SNPs remained after QC. Bi-allelic SNPs within coding regions were used for the majority of analyses in this release. There were 1,544,381 such SNPs in Pf8, which is 123,865 fewer than in Pf7. This reduction of bi-allelic coding SNPs was a result of the additional samples added to Pf8, and an increase in rare indel variants detected. This resulted in many positions previously SNP-only in Pf7 becoming a SNP-indel combination. The number of QC passed positions that were indel or SNP/indel combinations increased by 2,334,584 from Pf7 to a total of 5,077,522 in Pf8.

Based on the observation of an increase in SNP/indel combination positions, we also generated a SNP-only callset in Pf8. This includes all SNP positions already identified in the full callset, but removes all non-SNP variants, exposing previously masked SNPs in combined SNP/indel positions. A total of 10,821,552 SNPs are included in this callset, meaning there are SNPs at almost half of *P. falciparum* genomic positions (Supplementary Table 3). In the SNP-only callset, there are 2,565,888 bi-allelic coding SNPs passing QC available for analysis. The full callset was used for the analyses presented in this paper. Both the full and SNP-only callsets are available at https://www.malariagen.net/resource/36.

### Population structure

We investigated the global population structure of the Pf8 dataset using ten previously defined major sub-populations ^7^. These were previously defined based on geographic and genetic structure in the Pf7 release. A pairwise genetic distance matrix, generated from bi-allelic coding SNPs passing QC, was used to generate a neighbour joining tree (NJT) and principal coordinate analysis (PCoA) (Supplementary Figure 2). These investigations revealed that the ten major sub-populations are still broadly applicable within Pf8.

In Africa, four major sub-populations are present. These are West Africa (AF-W, 13,656 samples), Central Africa (AF-C, 1,549 samples), Northeast Africa (AF-NE, 470 samples), and East Africa (AF-E, 5,276 samples). With 8,298 additional, QC-passed samples added in Pf8, some shifts in population differentiation were identified relative to Pf7. Namely, samples in Africa showed less distinct clustering than in the Pf7 NJT ^7^. The majority of QC-passed samples new to Pf8 (6,074 / 8,298, 73%) were African, so shifts in the distinction between populations are more pronounced here than in other sub-populations, relative to Pf7. Weaker population structuring was especially evident between AF-C and other African sub-populations in Pf8 than in Pf7. Based on the NJT, several Central African samples appeared to cluster with East African samples, while a few clustered with West African samples. The Northeast African clade was also less distinct from East Africa than in Pf7.

Asian populations retained a more similar NJT topology to Pf7, although fewer new Pf8 samples were assigned to these populations. In South Asia, there were 246 samples in the eastern part (AS-S-E) and 1,730 samples in the far-eastern part (AS-S-FE). In the western part of Southeast Asia (AS-SE-W), Pf8 contains 2,262 samples, while in the eastern side (AS-SE-E), Pf8 contains 7,139 samples. The sub-population covering the Oceanian island of New Guinea (OC-NG) has 384 samples. Pf8 includes 283 samples classified within the South America (SA) sub-population.

An analysis of the clonality of samples via *F _WS_* scores revealed broadly the same sub-population-level trends as in Pf7 (Supplementary Figure 3). Samples from South America, western and eastern Southeast Asia, and Oceania were the most clonal, with the majority of samples having very high *F _WS_* scores > 0.95. The median *F _WS_* scores of these populations were 0.998, 0.997, 0.996, and 0.998, respectively. African sub-populations showed the lowest clonality, as expected for a region with high transmission. Median *F _WS_* scores were 0.974 in West Africa, 0.958 in Central Africa, and 0.951 in East Africa, with more samples extending to a lower *F _WS_* score than in other sub-populations. In contrast to Pf7, Northeast Africa showed higher sample clonality levels than in South Asia (eastern and far eastern). The median *F _WS_* for Northeast Africa was 0.996, while it was 0.992 in AS-S-E and 0.994 in AS-S-FE.

### Copy number variation

We developed a pipeline to detect amplifications of genes *gch1, crt, mdr1* and *plasmepsin 2/3*, and deletions of genes *hrp2* and *hrp3*. Similar to Pf7, we used a method based on GATK GermlineCNVCaller (gCNV) which can correct for the higher variation in coverage across the genome introduced by selective whole genome amplification (sWGA), before integrating the outputs with breakpoints-based evidence. Compared to Pf7, we modified the coverage-based method in such a way that the process became fully automated to allow greater reproducibility (Supplementary Methods, Supplementary Table 4).

**Figure 3.**
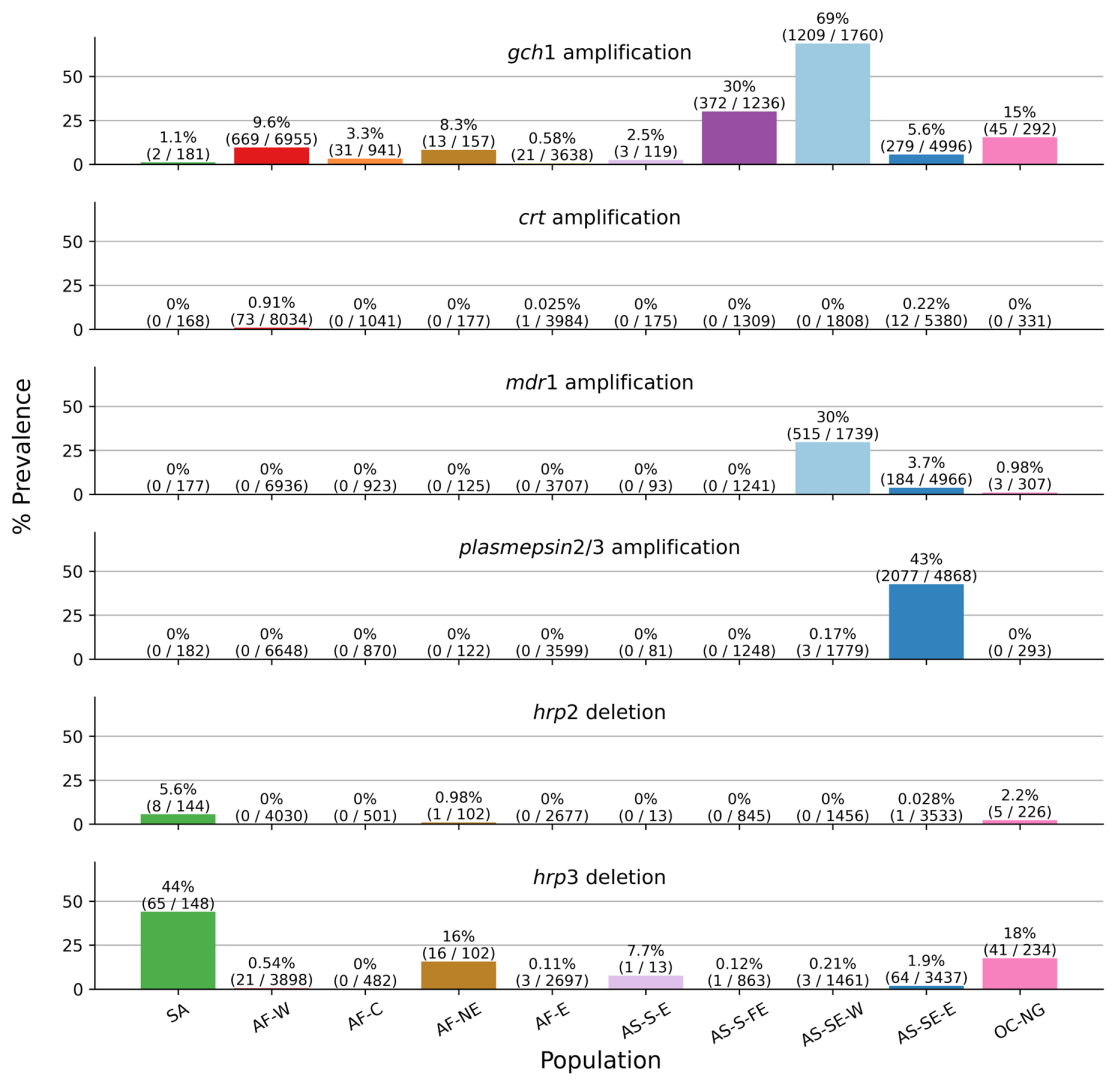
Population-level overview of the prevalence of copy number variations. Each row shows a series of bars indicating the percentage prevalence of one of the six copy number variations called in Pf8 in each of the ten colour-coded populations (colours are as per Figure 1). Each bar comprises the percentage prevalence, as well as the number of samples with the CNV (as the numerator) and the total number of samples with a non-missing CNV call (as the denominator).

Amplifications of the gene *gch1* have previously been linked with selection, likely driven by use of antifolates ^22^, though these have not previously been assessed in large global population studies. There is heterogeneity in the prevalence of *gch1* amplification across populations with a stark contrast between the western (69%) and eastern (5.6%) parts of Southeast Asia and also lower levels in east Africa compared to other parts of the continent ( Figure 3). In both parts of Southeast Asia, *gch1* amplifications appear to have decreased in recent years, whereas in west Africa, levels appear to be increasing slowly over time (Supplementary Figure 4A).

Examples of amplifications of *crt* have previously been reported ^23^. While there have been no reported associations of these duplications with any phenotypes of interest, it is worth monitoring them due to the key role of *crt* in resistance to various antimalarial drugs. *crt* amplifications are at 0.91% prevalence in west Africa, but also seen as rare cases in east Africa and the eastern part of Southeast Asia. The fact that most of these amplifications are seen in west Africa, and that there is little evidence of resistance to antimalarial drugs arising first in west Africa suggests these amplifications might not be driven by drug resistance, but further exploration of the phenotypic effect of *crt* amplifications are warranted.

Overall, levels of *hrp2* and *hrp3* deletions remained low. We saw no new *hrp2* deletions with respect to Pf7, and only a small number of new *hrp3* deletions. As in Pf7, we determined all breakpoints for *hrp2* and *hrp3* by manual inspection of reads. This revealed two new telomere healing associated breakpoints in samples from the Gambia and in Ghana (Supplementary Figure 5). It is worth noting that the majority of samples included in Pf8 originated from RDT-positive patients, therefore there could be biases.

### Drug resistance

We assessed the prevalence of known drug resistance markers associated with resistance to frontline antimalarial drugs. Using established genotyping heuristics ^7^, samples were classified as ‘Resistant’, ‘Sensitive’ or ‘Undetermined’ to ten major antimalarials and combination therapies, based on the presence of known resistance mutations or copy number variants (see https://www.malariagen.net/resource/36 for details of the heuristics used). This enabled us to infer resistance prevalence across geographic populations (Supplementary Tables 5 and 6). Resistance calls for individual samples were highly concordant with those in Pf7 ^7^, with only eleven changes in inferred resistance status among overlapping samples and drug resistance markers (Supplementary Figures 6 and 7). It is important to note that the genotyping heuristics are based on current understanding of the relationships between molecular markers and drug resistance phenotypes. They are not intended to provide clinical diagnostics or interpretation.

**Figure 4.**
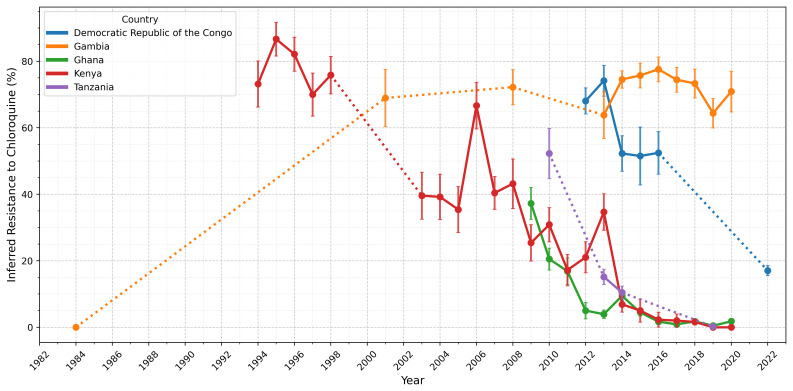
Inferred frequency of chloroquine resistance in five African countries. Points represent years for which more than 25 samples were available for a given country, while error bars show the standard error for a given year. Solid lines connect consecutive time points, while dotted lines indicate gaps greater than one year between adjacent time points, where years in between each had fewer than 25 samples. Notably, resistance has declined in Ghana, Kenya, and Tanzania from 20–60% to less than 5% since 2010. In the Democratic Republic of the Congo, resistance remained high until 2016 before decreasing, though there was only a single observation available after 2016. In contrast, Gambia is an outlier; after a sharp increase between 1984–2001, resistance has remained stable (60–80%) since around 2001.

Overall, the global distribution of inferred drug resistance frequencies remained consistent with previous releases (Supplementary Figure 8) ^7^, with the highest levels of resistance persisting in Southeast Asia (Supplementary Table 5). However, in African populations, we observed a modest decline in inferred chloroquine resistance over time ( Figure 4), likely reflecting reduced chloroquine use in these regions ^24^. This decline was evident in several individual African countries, including the Democratic Republic of the Congo, Ghana, Kenya and Tanzania. In Ghana, Kenya, and Tanzania, resistance frequencies fell sharply – from 20–60% to less than 5% after 2010. In the Democratic Republic of the Congo, resistance remained above 50% until 2016 before beginning to decline, though there was only a single observation available after 2016. In contrast, Gambia is an outlier; after a sharp increase between 1984–2001, resistance has remained stable between 60–80% since around 2001. These country-level changes led to an 8% reduction in the overall frequency of chloroquine resistance across the whole dataset compared to Pf7 (Supplementary Figure 8). However, as with Pf7, we observed substantial heterogeneity within geographic regions and over time, underscoring the need for careful interpretation of results and data from further locations and time points ^7^.

*mdr1* amplifications, associated with resistance to mefloquine, were generally more prevalent in the western part of Southeast Asia than the eastern part, with a steeper and earlier decline in the latter ( Figure 3, Supplementary Figure 4B). *gch1* and *mdr1* amplifications show striking similarity in their temporal trends in eastern Southeast Asia with prevalence around 50% in 2007 before rapidly declining to near-zero in subsequent years (Supplementary Figure 4A and 4B). *Plasmepsin2/3* amplifications, associated with resistance to piperaquine, were seen almost exclusively in the eastern part of Southeast Asia ( Figure 3). In this region, these amplifications were absent in 2007 but increased to 43%–74% between 2013 and 2019 before sharply declining to 2% in 2020 (Supplementary Figure 4C). These trends closely align with those recently reported by Verschuuren *et al.* ^25^.

## Additional tools

To enhance the accessibility and interpretability of Pf8, we have provided a suite of accompanying apps and analysis tools. The data exploration app ( https://apps.malariagen.net/apps/pf8/) describes the partner studies and people that have contributed samples and data to Pf8 and provides summary information about genetic markers of drug resistance in these samples.

The interactive web app, Pf-HaploAtlas ( https://apps.malariagen.net/pf-haploatlas) ^26^, now enables users to track spatiotemporal trends in amino acid haplotypes for any of the 4,952 core *P. falciparum* genes for all Pf8 samples.

We have updated the malariagen_data Python package, which provides methods allowing access to the Pf8 release data for custom analyses, such as accessing the sample metadata, variant calls and reference genome sequence and genomic features such as gene annotations. The package is available from PyPi at: https://pypi.org/project/malariagen_data/. All display items in this manuscript, in addition to updated versions of Pf7 display items, can be recreated and modified using free-to-use analysis guide notebooks, available at: https://malariagen.github.io/parasite-data/landing-page.html. All analysis notebooks can be executed interactively and for free within Google Colaboratory. The NextFlow pipeline used for calling short variants (SNPs and short indels) can be found at https://github.com/malariagen/malariagen-pf8-snp-indel-calling. The CNV calling pipeline can be found at https://github.com/malariagen/malariagen-pf8-cnv-calling.

## Concluding remarks

The release of Pf8 reflects MalariaGEN’s commitment to sequence and analyse *P. falciparum* parasites in order to understand and combat malaria. This release contains curated sequence data and genotypes for over 33,000 samples sequenced at the Wellcome Sanger Institute (WSI). Despite this very large data resource, there are still many areas of the malaria-endemic world for which there is limited data, for example central Africa, and there are still only a small number of regions for which there is longitudinal data spanning many years. For future releases, in addition to further samples sequenced at WSI, MalariaGEN are committed to working in partnership with global data generators willing to contribute to continue to grow this important, open access data resource.

We believe this resource contains the most samples of any other open data set of eukaryotic genome variation. Importantly, in addition to the genomic data, Pf8 contains rich metadata on date and place of sample collection making it possible to perform spatial and longitudinal analyses. We consider Pf8 to be a crucial foundational resource - not only for the malaria research community but also for broader studies on pathogen evolution and genomic evolution as a whole.

## Display items

**Table 1.** Breakdown of Pf8 samples by geography. Sites are divided into ten populations. Note that a) samples from Kisumu in western Kenya have been assigned to the Africa - Northeast (AF-NE) population, whereas samples from Kilifi in southern Kenya have been assigned to the Africa - East (AF-E) population, b) samples from Odisha and West Bengal in India to the west of Bangladesh have been assigned to the Asia - South Asia - West (AS-S-E) population, whereas samples from Tripura in India to the east of Bangladesh have been assigned to the Asia - South Asia - East (AS-S-FE) population and c) samples from Ranong and Tak in western Thailand have been assigned to the Western Southeast Asia (AS-SE-W) region, whereas samples from Ubon Ratchathani and Sisakhet in eastern Thailand have been assigned to the Eastern Southeast Asia (AS-SE-E) region. Unverified identity refers to samples with unverified or incomplete sample collection information (including lab strains and samples from returning travellers).

Region	Country	Admin level 1	Sequenced samples	Analysis set samples
**South America (SA)**	**Honduras**	**Francisco Morazan**	8	0
	**Peru**	**Loreto**	106	85
	**Colombia**	**Cauca**	146	123
		**Choco**	3	3
		**Nariño**	7	6
		**Norte de Santander**	8	5
		**Valle del Cauca**	3	3
	**Venezuela**	**Bolivar**	2	2
**Africa - West (AF-W)**	**Gambia**	**Banjul**	61	55
		**North Bank**	252	183
		**Upper River**	1,121	646
		**Western**	564	492
	**Senegal**	**Dakar**	93	91
		**Sedhiou**	62	60
	**Guinea**	**Faranah**	60	37
		**Nzerekore**	139	112
	**Mauritania**	**Guidimaka**	23	21
		**Hodh ech Chargui**	40	32
		**Hodh el Gharbi**	41	39
	**Côte d'Ivoire**	**Abidjan**	71	71
	**Mali**	**Bamako**	215	209
		**Kayes**	379	250
		**Koulikoro**	1,399	658
		**Mopti**	9	8
		**Segou**	265	71
		**Sikasso**	161	57
	**Burkina Faso**	**Haut-Bassins**	58	56
	**Ghana**	**Ashanti**	452	391
		**Brong Ahafo**	69	50
		**Central**	686	309
		**Eastern**	110	74
		**Greater Accra**	474	435
		**Upper East**	4,821	3,605
		**Volta**	41	41
	**Benin**	**Atlantique**	57	45
		**Littoral**	277	100
	**Nigeria**	**Adamawa**	72	37
		**Cross River**	73	16
		**Delta**	31	13
		**Federal Capital Territory**	5	0
		**Kano**	37	22
		**Kebbi**	71	25
		**Kwara**	8	5
		**Lagos**	188	129
		**Ogun**	96	38
		**Ondo**	56	29
		**Osun**	55	15
		**Oyo**	611	71
	**Gabon**	**Wouleu-Ntem**	59	55
	**Cameroon**	**Sud-Ouest**	294	261
**Africa - Central (AF-C)**	**Democratic Republic of the Congo**	**Kinshasa**	1,549	1,212
**Africa - Northeast (AF-NE)**	**Sudan**	**Al Jazirah**	17	1
		**Blue Nile**	128	1
		**Kassala**	41	18
		**Khartoum**	154	85
		**North Kordofan**	16	10
	**Uganda**	**Apac**	15	10
	**Ethiopia**	**Amhara**	15	10
		**Oromia**	19	12
		**Southern Nations, Nationalities and Peoples**	1	0
	**Kenya**	**Kisumu**	64	62
**Africa - East (AF-E)**	**Kenya**	**Kilifi**	2,078	1,872
	**Malawi**	**Chikwawa**	629	254
		**Zomba**	52	34
	**Tanzania**	**Kagera**	61	52
		**Kigoma**	199	143
		**Lindi**	79	65
		**Morogoro**	34	32
		**Pwani**	447	436
		**Tanga**	324	297
	**Mozambique**	**Cabo Delgado**	171	142
		**Gaza**	171	95
		**Inhambane**	117	99
		**Maputo**	603	417
		**Sofala**	65	23
		**Tete**	128	103
		**Zambezia**	93	85
	**Madagascar**	**Fianarantsoa**	1	1
		**Mahajanga**	24	23
**Asia - South - East (AS-S-E)**	**India**	**Odisha**	124	93
		**West Bengal**	122	95
**Asia - South - Far East (AS-S-FE)**	**India**	**Tripura**	72	62
	**Bangladesh**	**Chittagong**	1,658	1,315
**Asia - Southeast - West (AS-SE-W)**	**Myanmar**	**Bago**	124	89
		**Kachin**	28	26
		**Kayin**	768	645
		**Mandalay**	120	114
		**Rakhine**	19	9
		**Sagaing**	93	38
		**Shan**	65	30
		**Tanintharyi**	51	50
	**Thailand**	**Ranong**	27	20
		**Tak**	967	867
**Asia - Southeast - East (AS-SE-E)**	**Thailand**	**Sisakhet**	152	97
		**Ubon Ratchathani**	11	10
	**Laos**	**Attapeu**	538	444
		**Champasak**	282	244
		**Salavan**	240	214
		**Savannakhet**	891	783
		**Sekong**	43	38
	**Cambodia**	**Battambang**	65	51
		**Koh Kong**	5	5
		**Pailin**	286	187
		**Preah Vihear**	567	457
		**Pursat**	671	459
		**Ratanakiri**	420	357
		**Stueng Traeng**	268	232
	**Vietnam**	**Bac Lieu**	4	1
		**Binh Phuoc**	892	754
		**Binh Thuan**	11	0
		**Dak Lak**	311	300
		**Dak Nong**	116	112
		**Gia Lai**	815	756
		**Khanh Hoa**	95	82
		**Ninh Thuan**	215	85
		**Phu Yen**	94	89
		**Quang Nam**	95	76
		**Quang Tri**	52	47
**Oceania - New Guinea (OC-NG)**	**Indonesia**	**Papua**	133	119
	**Papua New Guinea**	**East Sepik**	166	149
		**Madang**	55	44
		**Milne Bay**	30	29
**Unverified identity**			330	0
**Total**			33,325	24,409

## Methods

### Sample collection

Most data in Pf8 are derived from blood samples, collected from patients with *P. falciparum* malaria. The exceptions are samples from studies 1041-PF-US-FERDIG, 1042-PF-HN-RANFORD-CARTWRIGHT, 1043-PF-GB-RAYNER, 1104-PF-LAB-WENDLER and 1153-PF-Pf3KLAB-KWIATKOWSKI, which are derived from cultured lab strains. Blood samples were collected with informed consent from patients, or their parents/guardians. Appropriate ethical approval was provided for each location in which samples were collected, please see the Consent section for further details. Genomic DNA was extracted from blood samples according to methods previously described ^27, 28^. Samples with sufficient DNA quantity and proportion of human DNA were progressed to whole genome sequencing (WGS) using Illumina technologies.

### Variant calling

A detailed summary of bioinformatics methods used in the creation of Pf8 can be found in the Supplementary Materials. All reads not aligning to a human reference genome were mapped with BWA mem version 0.7.17 ^29^ to the 3D7 v3 *P. falciparum* reference genome. Samtools version 1.13 ^30^ produced sorted and corrected BAM files in which duplicates were marked using GATK version 4.2.5.0 MarkDuplicates ^31^. Base Quality Score Recalibration (BQSR) was performed with GATK using all variants which passed quality filters in Pf7 to train the model. All lanes of sequencing data per sample were merged, creating a single sample-level BAM file for each of the 33,325 samples.

Variant calling was performed for each chromosome in two stages, firstly with GATK HaplotypeCaller for each individual sample and then with GATK GenotypeGVCFs to jointly call variants across the entire dataset. GATK Variant Quality Score Recalibration (VQSR) was implemented for SNPs and indels. Variants with a VQSLOD score ≤ 2 were failed. Variant annotations were added using snpEff (version 5.0e) ^32^ and bcftools v.1.13 ^33^. Any variant not in the core region of the genome failed to pass filtration. Filtered, all-sample, chromosomal VCF files were concatenated using bcftools concat before conversion to zarr format using zarr version 2.18.1 ^34^ and scikit-allel (version 1.3.8) ^35^. A snp-only callset was also created by using bcftools to filter out all non-snp variants from VCF files, then converting to a zarr file as above.

### Quality control

To ensure only *P. falciparum* samples were included in the release, we used GeneticReportCard ( https://github.com/malariagen/GeneticReportCard) and custom Python scripts to genotype samples at six mitochondrial loci capable of discriminating between six *Plasmodium* species. A final analysis set of 24,409 samples was created by assigning any sample with an unverified identity, a species other than *P. falciparum*, replicate samples, samples with <50% of their genome callable at 5X, and samples with >200 singleton SNPs as QC-fail.

### Genetic diversity and structure

Mean pairwise genetic distances were calculated between all samples, based on the non-reference allele frequency for bi-allelic SNPs, in coding regions, passing quality filters. Using the distance matrix, we checked the suitability of population assignments as determined in Pf7 ^7^. Population structuring was investigated amongst QC+ samples using a neighbour-joining tree (NJT) and principal coordinates analysis. Samples were coloured according to the same ten populations as defined in Pf7: South America, West Africa, Central Africa, Northeast Africa, East Africa, eastern South Asia, far-eastern South Asia, western Southeast Asia, eastern Southeast Asia, and Oceania. Clonality of QC+ samples was determined via calculation of *F _WS_* scores as previously described ^27^.

### Copy number variation calling

Gene amplification calls were made for four genes ( *mdr1*, *dhps*, *dhfr*, and *plasmepsin2/3*), and gene deletion calls were made for two ( *hrp2* and *hrp3*). The sequencing coverage of each sample was compared with other samples, to determine where the coverage deviated significantly enough to warrant calling a copy number variation using hidden Markov models (HMMs) from GATK tools (v4.5.0.0). The raw output files from the GATK functions were processed and analysed using Python to generate 6 CNV genotype calls for each QC-passed sample. For gene amplification calls, the genotype values could take one of three values: missing/uncallable (-1), not amplified (0), or amplified (1). In mixed samples, at least 50% of the parasites must contain an amplification for it to have been called as 1 (i.e., copy ratio must have exceeded 1.5). For gene deletion calls, the genotype values could be one of three values: missing/uncallable (-1), not deleted (0), or deleted (1). A small subset of the coverage-based calls were manually curated, before all the curated coverage-based calls was combined with breakpoints-based evidence to produce the final CNV calls. Similarly to Pf7, we calculated the proportion of face-away read pairs as a percentage of all reads within regions of known tandem duplication breakpoints, and if the proportion exceeded 2.5%, the breakpoints-based call would be 1 for the gene amplification calls. The breakpoints-based calls were then logically integrated with the coverage-based calls to produce a final set of CNV calls. For gene deletion calls, all samples with a coverage-based call of 1 were manually inspected on IGV for signs of breakpoint evidence for their final CNV call.

A code repository detailing the entire process and allowing others to perform CNV calls for their own samples can be found at https://github.com/malariagen/malariagen-pf8-cnv-calling. Detailed methods can be found in Supplementary Methods.

### Drug resistance

To infer drug resistance phenotypes, we used a heuristic framework that associates specific genetic variants in key loci (e.g., *crt, mdr1, dhfr, dhps,* and *kelch13*) with ‘Resistant’, ‘Sensitive’, or ‘Undetermined’ status for major antimalarial drugs. Classification criteria were based on current literature regarding point mutations and copy number variants (CNVs) (for details, see https://www.malariagen.net/resource/36). Each sample’s coding sequence at these loci was inferred by comparing variants from the VCF GT field to the 3D7 reference sequence, translating the resulting sequence to amino acids, and determining the presence of alleles linked with resistance.

## Consent

The following local and institutional committees gave ethical approval for the partner studies with new samples included in this data release:

Bangladesh Medical Research Council (BMRC), Bangladesh, Ministry of Health National Ethics Committee on Health Research, Cambodia; National Research Ethics Review Committee of Ministry of Science and Higher Education, Ethiopia; Ghana Health Service Ethics Review Committee, Ghana; Navrongo Health Research Centre Institutional Review Board, Ghana; Noguchi Memorial Institute of Medical Research, University of Ghana Institutional Review Board, Ghana; KEMRI Scientific and Ethics Review Unit (SERU), Kenya; Pharmacy and Poisons Board (PPB) Expert Committee on Clinical Trials (ECCT), Kenya; National Ethics Committee for Health Research (NECHR), Lao People's Democratic Republic; University of Khartoum Institute of Endemic Diseases Research Ethics Committee, Sudan; College of Medicine Research and Ethics Committee, Malawi; Comité d'Ethique des Facultés de Médecine/d'Odontostomatologie et de Pharmacie, Université des Sciences, des Techniques et des Technologies, de Bamako, Mali; Institutional Review Board (CIBS) at Manhiça Health Research Centre (CISM), Mozambique; Medecins sans Frontiere (MSF) Ethics Review Board (ERB); National Committee for Bioethics in Health of Mozambique (CNBS), Mozambique; Department of Medical Research (Lower Myanmar), Myanmar; College of Medicine, University of Lagos, Nigeria; Cross River State Health Research Ethics Committee, Cross River State Ministry of Health, Calabar, Nigeria; Federal Medical Centre, Yola, Nigeria; Nigerian Institute of Medical Research Institutional Review Board, Lagos, Nigeria; Comite Institucional de Ética en Investigacion de la Universidad Peruana Cayetano Heredia, Peru; US Naval Medical Research Unit SOUTH (NAMRU SOUTH) Institutional Review Board, Lima, Peru; US Naval Medical Research Unit SOUTH Institutional Review Board, Lima, Peru; Muhimbili University of Health and Allied Sciences Ethical Review Board, Tanzania; National Institute for Medical Research, Tanzania; University of Khartoum Institute of Endemic Diseases Research Ethics Committee, Sudan; Ethics Committee of the Faculty of Tropical Medicine, Mahidol University, Thailand; The Gambia Government/MRC Joint Ethics Committee, Banjul, The Gambia; Oxford Tropical Research Ethics Committee, Oxford, UK; Institute of Malariology-Parasitology-Entomology, Ho Chi Minh City, Vietnam; and Institute of Malariology-Parasitology-Entomology, Quy Nhon, Vietnam.

For information regarding the local and institutional committees that gave ethical approval for the partner studies with previously published samples included in this release, please see Pf7 ( https://wellcomeopenresearch.org/articles/8-22/v1).

## Disclaimer

The views expressed in this article are those of the authors and do not necessarily reflect the official policy or position of the Department of the Navy, Department of Defense, nor the U.S. Government.

## Data Availability

This project contains the following underlying data that are available as an online resource: https://www.malariagen.net/resource/36. Sample provenance and sequencing metadata: sample information including partner study information, location and year of collection, ENA accession numbers, and QC information for 33,325 samples from 34 countries. CNV calls: amplification calls for genes CRT, GCH1, MDR1 and PM2_PM3, and deletion calls for HRP2 and HRP3. Tandem duplication breakpoints: genomic coordinates of breakpoints used for faceaway read-based calling. Measure of complexity of infections: characterisation of within-host diversity (Fws) for 24,409 QC pass samples. Drug resistance marker genotypes: genotypes at known markers of drug resistance for 24,409 samples, containing amino acid and copy number genotypes at six loci: *crt*, *dhfr*, *dhps*, *mdr1*, *kelch13, plasmepsin 2–3*. Inferred resistance status classification: classification of 24,409 QC pass samples into different types of resistance to 10 drugs or combinations of drugs and to RDT detection: chloroquine, pyrimethamine, sulfadoxine, mefloquine, artemisinin, piperaquine, sulfadoxine- pyrimethamine for treatment of uncomplicated malaria, sulfadoxine- pyrimethamine for intermittent preventive treatment in pregnancy, artesunate-mefloquine, dihydroartemisinin-piperaquine, *hrp2* and *hrp3* gene deletions. Drug resistance markers to inferred resistance status: details of the heuristics utilised to map genetic markers to resistance status classification. Reference genome: the version of the 3D7 reference genome FASTA file used for mapping (PlasmoDB-54-Pfalciparum3D7-Genome.fasta). Annotation file: the version of the 3D7 reference annotation general feature format (gff) file used for genome annotations (PlasmoDB-55_Pfalciparum3D7.gff.gz). Genetic distances: Genetic distance matrix comparing all 33,325 samples (NumPy array). SNP-only genetic distances. Genetic distance matrix comparing all 33,325 samples using SNP-only call set (NumPy array). Short variants genotypes: Genotype calls on 12,493,205 SNPs and short indels in all 33,325 samples from 34 countries, available both as variant call format (VCF) and Zarr files. SNP-only genotypes: Genotype calls on 10,821,552 SNPs in all 33,325 samples from 34 countries, available both as VCF and Zarr files. CRAM files: Compressed sequencing data files for all 33,325 samples gVCF files: genomic VCF files containing both variant and non-variant regions for all 33,325 Pf8 samples Supplementary materials (extended methods, display items, and contributing study details) are available via Figshare at the following DOI: 10.6084/m9.figshare.29153447. Data are available open access under the terms of the Creative Commons Attribution 4.0 International license (CC-BY 4.0).
