# Supplementary material for: Pf8: an open dataset of Plasmodium falciparum genome variation in 33,325 worldwide samples
Source: Wellcome Open Res. 2025 Jun 30;10:325. [Version 1] doi: 10.12688/wellcomeopenres.24031.1 (PMC12627940; doi:10.12688/wellcomeopenres.24031.1)
Supplement: Peer review history

# Reviewer report: Reviewer response for version 1

**DOI:** 10.21956/wellcomeopenres.26513.r137757

**Published:** 2025-12-19

**Authors:** Wim L Cuypers

**Affiliations:** 1 University of Antwerp, Antwerp, Flanders, Belgium

The Pf8 data release represents an impressive collaborative achievement and a milestone in open pathogen genomics. I commend the authors for their commitment to transparent science and for delivering a high-quality, rigorously curated resource. The bioinformatics methods are solid, with thoughtful handling of species confirmation, variant calling, and CNV detection, supported by well-documented pipelines and reproducible workflows.

 I have no major comments, just some suggestions for the manuscript that may also inform the next version.

 The manuscript remains within scope as a data note, yet the scale, temporal depth, and geographic breadth naturally raise forward-looking questions, particularly about addressing remaining surveillance blind spots. A brief discussion of how future sampling efforts could fill these gaps would be appreciated.

 The dataset critically depends on samples from low- and middle-income countries. A section reflecting on how this effort can contribute to capacity strengthening, through training, field-deployable sequencing, or local data interpretation support, would strengthen the manuscript’s positioning (or the next release) within equitable global health science.

 Clarifying how patient travel history was obtained and how the ‘country’ field is defined could benefit researchers. Is the ‘country’ field always the the country of exposure of the patients? And otherwise, the 'unverified' label is assigned?

 The Pf-HaploAtlas is an exciting tool with clear potential for use in tracking lineages through haplotypes of drug resistance genes or antigens. The authors could expand on how multi-locus haplotype profiles might serve a role similar to sequence types or strain clusters in bacterial surveillance.

 What do the error bars represent exactly in Figure 4? Confidence intervals?

 The CNV and SNV workflows are a mix of Nextflow pipelines and Jupiter notebooks. While modularity and interactivity have clear benefits, the reproducibility and automation of the pipeline could be improved by integrating the notebooks into a unified NextFlow workflow with optional prompts.

 Mapping to 3D7 is standard, but with over half the genome positions varying across the dataset, limitations of a single reference become apparent. Acknowledging the potential of pan-genome or graph-based reference frameworks, especially for highly diverse regions like the African continent, would be forward-looking. The dataset and methods laid out in this release could form the foundation for such efforts.

 Similarly, more nuanced phylogenetic reconstruction (e.g., using RAxML ) could help resolve structure within sub-populations. This might be of interest for future updates or example analyses.

Are sufficient details of methods and materials provided to allow replication by others?

Yes

Is the rationale for creating the dataset(s) clearly described?

Yes

Are the datasets clearly presented in a useable and accessible format?

Yes

Are the protocols appropriate and is the work technically sound?

Yes

Reviewer Expertise:

Microbial genomics, bioinformatics, infectious diseases, antimicrobial resistance.

I confirm that I have read this submission and believe that I have an appropriate level of expertise to confirm that it is of an acceptable scientific standard.

# Reviewer report: Reviewer response for version 1

**DOI:** 10.21956/wellcomeopenres.26513.r137754

**Published:** 2025-11-18

**Authors:** Daniel Jeffares

**Affiliations:** 1 University of York, York, England, UK

This article describes the Pf8 resource of *Plasmodium falciparum* genomes. This is an extremely valuable resource generated through considerable effort by many researchers. The data are highly valuable for malaria studies, for genomic epidemiology more broadly, and for evolutionary genetics. While the main focus is to describe the current release of the data (compared to the previous Pf7 resource), the article contains several highlights that demonstrate the value of the resource, including the global prevalence of copy‑number variations in *P. falciparum* that may be related to drug resistance, and time‑series information showing the rise and fall of chloroquine‑resistance mutations. Despite some minor limitations, the article serves the purpose of describing the resource well.

 Strengths of this article and resource are:

 - the rationale for creating the data is clear, given the continued burden of malaria

 - the data for this parasite are centralised

 - the documentation of methods, including data‑access tools, is generally very good

 The limitations, which are relatively minor, are as follows:

 - The description of sample and SNP call counts is unnecessarily complex. Although 33,000 samples were sequenced, not all met quality criteria, leaving approximately 24,000 usable samples. The resulting SNP counts are accordingly complicated.

 - The claim that this resource is the largest open dataset of genome variation in any eukaryotic species is not strictly correct. The human variation dataset gnomAD v4.1.0 contains 76,215 human genomes, which is more than twice as large in terms of samples and many times larger in terms of SNPs (see: https://gnomad.broadinstitute.org/data).

 - While the documentation of methods is generally very good, there appears to have been a proliferation of tools and websites. For Pf9, a 'spring clean' of resources might be appropriate.

 - Datasets could be made available via simpler methods, such as FTP or wget; anyone analysing these data will be comfortable using those tools.

 Nevertheless, this Pf8 resource of *Plasmodium falciparum *genomes is extremely valuable and the article describes the data well enough to serve the purpose.

Are sufficient details of methods and materials provided to allow replication by others?

Yes

Is the rationale for creating the dataset(s) clearly described?

Yes

Are the datasets clearly presented in a useable and accessible format?

Yes

Are the protocols appropriate and is the work technically sound?

Yes

Reviewer Expertise:

Molecular evolution, population genetics/genomics and quantitative genetics with a focus on small eukaryotic genomes, including parasites.

I confirm that I have read this submission and believe that I have an appropriate level of expertise to confirm that it is of an acceptable scientific standard.

# Reviewer report: Reviewer response for version 1

**DOI:** 10.21956/wellcomeopenres.26513.r127191

**Published:** 2025-08-27

**Authors:** Qixin He

**Affiliations:** 1 Purdue University, West Lafayette, Indiana, USA

The authors have curated a new genomic database for P. falciparum, and have expanded the size from the previous pf7 by 60%. In addition to SNP callings, they now include CNV for important drug-resistant genes and rapid diagnostic test failures. This open database will help immensely in studying disease dynamics, resistance evolution and vaccine design. I consider this a significant contribution to the research of genomic epidemiology in malaria. 

 I have a minor comment regarding population structure analysis for the authors to address. The authors described a total of ten populations globally, including four sub-populations in Africa and four in South Asia. However, on the PCOA plot or the NJ plot in Supplementary Figure 2, African populations are difficult to distinguish from each other, and those from Southeast Asia to a lesser extent. As the parasites are sexual, I don't think the NJ method is appropriate for differentiating populations. Similarly, since African samples include many infections with MOI > 1, they may blur the signals of population differentiation in the PCOA analyses. I recommend that the authors refer to these populations as geographically defined and discuss their genetic distance or overlapping clustering from these analyses, rather than using this evidence to define or delineate populations. Limitations of the analysis should also be briefly mentioned.

Are sufficient details of methods and materials provided to allow replication by others?

Yes

Is the rationale for creating the dataset(s) clearly described?

Yes

Are the datasets clearly presented in a useable and accessible format?

Yes

Are the protocols appropriate and is the work technically sound?

Yes

Reviewer Expertise:

malaria infectious disease modeling; molecular epidemiology; population genomics.

I confirm that I have read this submission and believe that I have an appropriate level of expertise to confirm that it is of an acceptable scientific standard.
